# 

**Published:** 1918-04-13

**Authors:** 


### CONTENTS.

PAGE

**National Necessities and Sacrifices** ... ... 21

**Phrases and Realities** ... ... ... ... ... 22

**Hospital and Institutional News** ... ... ... 23

America's Gift—The Royal Free Hospital—The Future of Golders Green—Segregation for Nerve Cases—The R.A.M.C. in the Great Battle—Convalescents in Billets—The American Ambulance Train—Deaths in Cottage Hospitals—Hospital Activity in Belfast—The College of Ambulance—Death of a Former Hospital Secretary—The Spread of Trench Fever—The Red Cross in Holland—Complaints Against a Medical Board—Some St. Dunstan's Surprises—The March *Searchlight*—Saccharin and the Food Controller—This Week's Drug Market.

**London's Institutional Medical Relief** ... ... 27

Proposals for its Reform.

**A Remarkable Organisation** ... ... ... ... 28

**Lord Lister** ... ... ... ... ... ... ... 29

XII. His Triumph, Complete and Universal.

PAGE

**St. Paul's Cathedral** ... ... ... ... ... ... 31

Memorial Service for Nurses who have Fallen in the War.

**Nursing Affairs in Ireland** ... ... ... ... 32

The Absence of Esprit de Corps.

**Nursing Progress and its Developments** ... 33

XII. The Local Aims of Local Centres.

Round the Hospitals.

The Heroines' Roll of Honour—Uniform Without Recognition or Protection—Leicester Solid for the College—College Nominations by April 17—Colonial and American Nursing Points.

**Food in War-Time** ... ... ... ... ... 37

**Hospital Architecture and Construction** ... 38

King Edward VII.'s Hospital, Cardiff.

**Gifts and Bequests** ... ... ... ... ... ... 39

**Matrons' and Sisters' Appointments** ... ... 40

**The Editor's Letter-Box** ... ... ... ... 40

**The Institutional Worker** ... ... (Suppl.) 1

